# *Fusarium oxysporum* f. sp. *phaseoli* genetic variability assessed by new developed microsatellites

**DOI:** 10.1590/1678-4685-GMB-2019-0267

**Published:** 2020-05-29

**Authors:** Graziéle R. Sasseron, Luciana L. Benchimol-Reis, Juliana M.K.C. Perseguini, Jean Fausto C. Paulino, Miklos M. Bajay, Sérgio A.M. Carbonell, Alisson F. Chiorato

**Affiliations:** 1 Instituto Agrônomico (IAC), Centro de Pesquisa em Recursos Genéticos, Campinas, SP, Brazil.; 2 Universidade Tecnológica Federal do Paraná, Dois Vizinhos, PR, Brazil.; 3 Universidade do Estado de Santa Catarina (UDESC), Centro de Educação Superior da Região Sul da (CERES), Departamento de Engenharia de Pesca e Ciências Biológicas (DEPB), Laguna, SC, Brazil.; 4 Instituto Agrônomico (IAC), Centro de Análise e Pesquisa Tecnológica do Agronegócio dos Grãos e Fibras, Campinas, SP, Brazil.

**Keywords:** markers, common bean, simple sequence repeats, diversity

## Abstract

*Fusarium oxysporum* f. sp. *phaseoli* (*Fop*) J.B. Kendrich & W.C. Snyder is the causal agent of Fusarium wilt of common bean (*Phaseolus vulgaris* L.). The objective of this study was to develop microsatellite markers (SSRs) to characterize the genetic diversity of *Fop*. Two libraries enriched with SSRs were developed and a total of 40 pairs of SSRs were characterized. Out of these, 15 SSRs were polymorphic for 42 *Fop* isolates. The number of alleles varied from two to ten, with an average of four alleles per *locus* and an average PIC (Polymorphic Information Content) of 0.38. The genetic diversity assessed by microsatellites for *Fop* was low, as expected for an asexual fungus, and not associated with geographic origin, but they were able to detect enough genetic variability among isolates in order to differentiate them. Microsatellites are a robust tool widely used for genetic fingerprinting and population structure analyses. SSRs for *Fop* may be an efficient tool for a better understanding of the ecology, epidemiology and evolution of this pathogen.

## Introduction

*Fusarium oxysporum* f. sp. *phaseoli* (*Fop*), causal agent of fusarium wilt in common bean, is currently considered one of the most important bean diseases (Ramalho *et al.*, 2012). The *Fop* fungus is the main bean soil pathogen and has been established in all bean producing areas of Brazil. Losses caused by this disease have been increasing mainly in areas under successive and irrigated plantations (Toledo-Souza *et al.*, 2012). In addition to the lack of chemical control, this scenario is aggravated by the production of chlamydospores, fungal resistance structures that survive for many years in the soil, even in the absence of the host. For these reasons, the main alternative to control this disease is to obtain resistant cultivars (Cross *et al.*, 2000) that are easily adopted by producers and do not present environmental risks (Gonçalves-Vidigal *et al.*, 2013).

*Fusarium* species that cause vascular wilting are all classified as *Fusarium oxysporum* (Pereira, 2009), which forms a complex of soil fungi composed of patotypes classified into various *forma specialis*, based on pathogenic criteria. They are responsible for the disease in more than 100 plant species (Pantelides *et al.*, 2013). Each *forma specialis* group is pathogenic to specific plant group, demonstrating the degree of host specificity (Nelson *et al.*, 1983).

*Fusarium* *oxysporum* sp. *phaseoli* can penetrate an intact root tissue, but also penetrate into more developed parts of the root and hypocotyl tissues also occurs, usually through injury or natural openings (Paula Júnior *et al.*, 2006). *Fop* penetrates plants through the root system and colonizes the xylem, causing wilting, vascular discoloration, chlorosis, dwarfism and premature plant death (Nelson *et al.*, 1983).

In resistant plants, symptoms are few or less expressive; occluding material is observed in the xylem vessels of inoculated plants (Pereira *et al.*, 2013) and it has been associated to a delay in fungal colonization in the host.

In bean plants, the variability of physiological races of the *Fop* fungus has been studied by several authors (Ribeiro and Hagedorn, 1979a; Ribeiro and Hagedorn, 1979b; Salgado and Schwartz, 1993; Salgado *et al.*, 1995; Woo *et al.*, 1996; Ito *et al.*, 1997; Alves-Santos *et al.*, 2002a). Several physiological races are described, but all authors report that further studies are needed.

There is a preference for microsatellite markers or simple sequence repeats (SSRs) in contrast to other types of markers, since they use the agility of the PCR technique, are codominant and randomly scattered in the genome with a relatively high frequency as well (Karaoglu *et al.*, 2005). DNA sequences flanking microsatellites are generally conserved among individuals of the same species, or even between related species. These sequences are made up of one to six nucleotide repeats that occur naturally in the genome.

Microsatellites in fungi are more difficult to isolate and exhibit a lower polymorphism than in other organisms (Dutech *et al.*, 2007). Bogale *et al.* (2005) described nine SSR markers developed for the study of *Fusarium oxysporum.* According to Cruz *et al.* (2018) there is a positive correlation between Fusarium diversity and its virulence in common bean. A major drawback of this study was that they used only seven polymorphic microsatellites out of eighteen tested to build the clustering and sustain their conclusions of positive correlation between virulence and diversity. A limitation of the use of SSRs in *F. oxysporum* f. sp. *phaseoli*, in general, is that very few markers were developed for the study of this fungus. Developing SSR markers is very laborious and costly because it traditionally involves the screening of enriched genomic libraries (Zane *et al.*, 2002). Accurate identification and knowledge of the genetic diversity of pathogenic *Fusarium* *oxyporum* f. sp. *phaseoli* is important in the management of the disease and a large set of microsatellites in needed for providing full genome coverage.

## Material and Methods

For the present study, 42 pathogenic isolates of *F.oxysporum* f. sp. *phaseoli* were collected in different states of Brazil. The study of diversity and genetic structuring was carried out with the 42 *Fop* isolates, 3 isolates collected in the State of Goiás, 24 isolates collected in the State of São Paulo, 1 isolate collected in the State of Pernambuco, 10 isolates collected in the State of Minas Gerais, 2 isolates collected in the State of Santa Catarina and 2 isolates collected in the State of Paraná (Table 1).

**Table 1:** *Fusarium oxysporum* f. sp. *phaseoli* isolates with their respective origins and codes.

ID number	Isolate code	City	State	Isolation year
01	IAC 11205	Casa Branca	SP	1999
02	IAC 11299	Capão Bonito	SP	1999
03	IAC 11173	Angatuba	SP	1999
04	IAC 11257	Capão Bonito	SP	1999
05	IAC 11233	Capão Bonito	SP	1999
06	IAC 11472	Itararé	SP	1999
07	IAC 11018	Esp. Santo do Pinhal	SP	1999
08	IAC 11178	Taquarituba	SP	1999
09	IAC 11293	Tarumã	SP	1999
10	IAC 11848	Votuporanga	SP	2000
11	*FOP* 46 Embrapa	Belém de São Francisco	PE	2001
12	IAC 12802	Capão Bonito	SP	2003
13	IAC 14296	Itapetininga	SP	2009
14	IAC 14353	Campos Novos	SC	2010
15	IAC 14428	Esp. Santo do Pinhal	SP	2010
16	IAC 14435	Pindorama	SP	2010
17	IAC 14352	Campos Novos	SC	2010
18	IAC 14437	Pindorama	SP	2010
19	FOP 42	Santo Antônio de Goiás	GO	2011
20	FOP 48	Foz do Jordão	PR	2011
21	*FOP* UFV 05 (FOP Canaã)	Canaã	MG	2011
22	*FOP* Coimbra 04 - UFV	Coimbra	MG	2011
23	*FOP* UFV 06 (FOP Coimbra)	Coimbra	MG	2011
24	*FOP* UFV 01	Coimbra	MG	2011
25	*FOP* UFV 02	Coimbra	MG	2011
26	*FOP* UFV 03	Coimbra	MG	2011
27	*FOP* UFV 04	Coimbra	MG	2011
28	IAC 14564	Mococa	SP	2011
29	*FOP* Coimbra03 -UFV	Coimbra	MG	2011
30	*FOP* UFV08 (FOP UNAI- UFV)	Unaí	MG	2011
31	IAC 14685	Adamantina	SP	2012
32	IAC 14645	Unaí	MG	2012
33	IAC 14675	Campinas	SP	2012
34	IAC 14629	Cerqueira César	SP	2012
35	IAC 14655	Cerqueira César	SP	2012
36	IAC 14671	Queda do Iguaçu	PR	2012
37	IAC 14684	Votuporanga	SP	2012
38	IAC 14714	Paranapanema	SP	2012
39	*FOP* UFV09 (UFV Vianópolis)	Vianópolis	GO	2013
40	IAC *FOP* 03/13	Colina	SP	2013
41	IAC *FOP* 04/13	Votuporanga	SP	2013
42	IAC *FOP* 05/13	Alto Paraíso do Goiás	GO	2013

Among the 42 *Fop* isolates used, 29 isolates belong to the mycoteca of the Phytopathology Laboratory of the Grains and Fiber Centre of the Agronomic Institute (IAC, Campinas, SP), five isolates belong to the micro-library of the Phytopathology Laboratory of EMBRAPA Rice and Beans (Goiânia, GO), and eight isolates belong to the Phytopathology Laboratory of the Federal University of Viçosa - UFV (Viçosa, MG, Table 1). The fungi were maintained in PDA (potato-dextrose-agar) culture medium in a growth chamber incubator at 24 °C.

In this study, two monosporic isolates of the pathogen were classified into physiological races according to the classification system proposed by Alves-Santos *et al.* (2002b). The same classification system and races were also used in the evaluation of 26 common bean genotypes for resistance to *Fusarium oxysporum* f. sp. *phaseoli* by Azevedo *et al.* (2015). Regarding the origin of the isolates, the first one named IAC 11173 (ID:3) was characterized as being from the American race or race I and the second one, named IAC 11233 (ID:5), characterized as a Brazilian race or race II, obtained from common bean plants with characteristic symptoms of the disease, collected in the municipalities of Angatuba and Capão Bonito, in the State of São Paulo, respectively.

Isolates IAC 11848 (ID:10) and IAC 12802 (ID:12) were used by Silva *et al.* (2014) in phylogenetic studies from molecular analyses of partial sequences of the elongation factor gene (EF-1α gene) and ribosomal intergenic spacer region (IGS rDNA) of Brazilian *Fop* strains, where the polyplyletic origin of the strains within both special form types of *Fusarium oxysporum* (f. sp. *phaseoli* and f. sp. *vasinfectum*) was demonstrated.

Isolates 11205 (ID:01), 11299 (ID:02), 11173 (ID:03), 11257 (ID: 4), 11472 (ID: 6), 11178 (ID: 8), *Fop* 46 (ID:11), 14435 (ID: 16), *Fop* 42 (ID: 19), *Fop* UFV01 (ID: 24), *Fop* UFV02 (ID: 25), *Fop* UFV03 (ID: 26), *Fop* UFV04 (ID: 27) and 14629 (ID: 34) were used by Cruz *et al.* (2018) in a study of molecular diversity in *Fusarium oxysporum* f. sp. *phaseoli* of bean fields in Brazil.

Henrique *et al.* (2015) described the use of three Brazilian pathogenic isolates in the characterization of *Fop* 46 (ID: 11), *Fop* 48 (ID: 20) and *Fop* 42 (ID: 19) of *Fusarium oxysporum* f. sp. *phaseoli* identified as races 2, 3 and 6, respectively, which were previously classified by Wendland *et al.* (2012) using the methodology of Alves-Santos *et al.* (2002b).

Two enriched microsatellite libraries (adapted from Billotte *et al.*, 1999) were developed, one for race I (IAC 11173, American) and one for race II (IAC 11233, Brazilian) for *Fop*. Genomic DNA was extracted from fungi mycelium, according to the instructions of the Wizard Genomic DNA Purification kit (Promega). DNA was digested with an *Afa I* restriction enzyme (10 u/μL) (Invitrogen), followed by the ligation of the *Afa* 21 adapter fragments (5CTCTTGCTT ACGCGTGGACTA3) and *Afa* 25 (5TAGTCCACGCGT AAGCAAGAGCACA3). The enrichement process was performed by means of the hybridization of the probes conjugated with biotin, (CT)_8_ and (GT)_8_.

The fragments enriched for microsatellites were cloned into the plasmidial vector pGEM-T and the transformation to competent cells was performed using a protocol adapted from Avi Levy, 1991 (personal communication). For transformation, 100 μL of *E. coli* competent cell solution (JM109, Promega) was taken from the −80 °C freezer and placed on ice. Then 2 μL of the ligation and 8 μl of Transfobuffer (10X KCM and 10% PEG) were added, gently shaken, and left on ice for 30 min. After that, the solution was removed and kept at room temperature for 10 min. Then 450 μL of S.O.C. medium (Thermo Fisher Scientific) was added and incubated at 37 °C for 1 hour in a shaker at 225 rpm. Transformed cells were plated in solid LB medium containing ampicillin (50 mg/mL), 60 μL IPTG (24 mg/mL), 60 μL X-Gal (20 mg/mL). One volume of each (bacteria, X-Gal, IPTG) was placed on an opposite portion of the plate, spread with a Drigalsky handle until dry, and incubated overnight at 37 °C. The plates were then refrigerated for 1-2 h so that the colonies turned blue. Positive clones were selected using the β- galactosidase gene. The clones from each library (race I and race II) were sequenced in a 3730 DNA Analyzer (Applied Biosystems). The SSRs were designed using Primer3 Software.

The amplification reactions of the multiplex SSRs were performed in a final volume of 15 L, containing 6.5 μL PCR Master Mix 2x kit (Fermentas), 1 μL DNA (50ng) of each *Fop* isolate, and concentrations of the individual primer pairs (10 pmol each, Table 2), depending on the intensity of the amplified product. The amplifications were carried out in a thermocycler, with an initial denaturation step of the DNA at 94°C for 4 minutes, followed by 34 cycles at 94°C for 30 seconds, with an annealing temperature for 1 minute and extension of 72°C for 1 minute. At the end of the cycles, there was another extension at 72°C for 10 minutes.

**Table 2:** Characteristics (the 5’ end labeling fluorophores; F: the forward primer sequences; R: the reverse primer sequences; *T*a: annealing temperature; sizes of the amplified fragments in base pairs-bp) of the microsatellites (SSRs) developed for *Fusarium oxysporum* f. sp. *phaseoli* isolates.

SSR/ fluorophores	Primer sequences (5’-3’)	Motifs	Number of Alleles	TA (°C)	Sizes of the amplified Fragments (bp)	PIC	GenBank Accession number
FOP 01-B01 NED	F – CCGCCGATTTCCTTACCT	(AT)_9_	03	58	237-254	0.13	MK622886
	R - GCACCCTTCAAACCTCCA						
FOP 07-B01 PET	F - TGAGCGAATGGGAAGAGG	(GGAG)_5_	06	58	151-225	0.54	MK622887
	R - TGCCAAGGGAGTATCATTTC						
FOP 10-B01 PET	F – ATGGGATAGGCGGTTTGG	(TC)_3_/(AG)_3_	05	58	236-255	0.77	MK602310
	R - ACTTGGGGTTGAGCGTTG						
FOP 13-B01 6FAM	F - TTCTTGCTGATCGCCTCA	(GGCAT)_5_	02	59	152-167	0.37	MK602312
	R - CCTCCCCTGCAATAAGAGC						
FOP 15-B01 NED	F - CCGTCTTCATCTTGGCGTCTA	(AGAT)_4_	03	54	209-225	0.09	MK622888
	R - TATCTAAGGGGTGGGACGGAG						
FOP 16-B01 PET	F - CCGCGCTCTCGAGCAGGG	(ACT)_3_	02	54	202-205	0.35	MK602313
	R - TGCCGAAGTAAAAGCACGG						
FOP 20-B01 6-FAM	F - CCGTTTGGCGCAAGTT	(GA)_6_	10	54	148-188	0.70	MK622889
	R - CGAGCGGCTTGTCTTCA						
FOP 28-B01 PET	F - GTTGCGCTCCCCAATATG	(TC)_3_/(AT)_3_	02	56	204-208	0.09	MK622890
	R - CCTCCATTGCCATCCATTT						
FOP 35-B01 NED	F - GGCTGGTGGTTTCAAGAGAG	(CAC)_6_	06	58	210-236	0.40	MK622891
	R – CAAGGCTTCTTCACGGGTAA						
FOP 37-B01 NED	F - GCAACACAGGAGGTGGTA	(TGCT)_3_	04	54	180-192	0.29	MK622892
	R - CAAGGGTATTGGTGCTTG						
FOP 08-B02 6-FAM	F - ACAGTGGCTCGTGACTCCA	(CT)_3_(TA)_3_	06	59	222-240	0.38	MK602309
	R - TGTTGGCACAGAGGCAGA						
FOP 09-B02 NED	F - TGCAGCGATGAGATTGGA	(CCA)_3_	03	54	165-169	0.35	MK622893
	R - GCGTCATCTCGTGAATCG						
FOP 11-B02 VIC	F - AACAGCCGAAGCCGATG	(AG)_7_	07	59	259-305	0.62	MK602311
	R - TTCACCTTCCACTTGCACA						
FOP 18-B02 PET	F - AATGCGGCCACTGTGATT	(TCA)_3_/(ATC)_2_	04	59	151-164	0.33	MK602314
	R - GCAGAAGTTGCGGTTGTG						
FOP 23-B02 NED	F - CAGGGGCACAGTTCGGTA	(TC)_3_/(AT)_3_	04	59	190-196	0.35	MK622894
	R - ATCGGCTGGGACATGAAG						

For the automated analyzer genotyping, multiplex sets were formed, containing three to four microsatellite *loci* (1st multiplex: FOP 01-B01, FOP 10-B01, and FOP 13-B01; 2nd multiplex: FOP 07-B1, FOP 15-B01, and FOP 20-B01; 3rd multiplex: FOP08-B02, FOP11-B02, FOP18-B02, and FOP23-B02; the other primers were used alone). The selection of the oligonucleotides used in the multiplex assembling considered that the same primers do not have complementarity between their bases and the fluorescences had different colors. After assembling the multiplex system, the 5’ end labeling of the oligonucleotides was performed with the 6-FAM, NED, PET, and VIC fluorophores so that only the forward primer was labeled with a fluorophore. The samples used in the genotyping were prepared in a reaction containing 8.85 μL formamide (Applied Biosystems), 0.15 μL standard marker (LIZ 500®, Applied Biosystems) and 1 μL PCR product diluted 20 ×. The amplified fragments were genotyped on a 3730 DNA Analyzer automated sequencer (Applied Biosystems). Product analyses were performed using the Peak Scanner TM v.1 program (Applied Biosystems).

### Statistical Analysis

The PIC (*Polymorphism Information Content*) was calculated based on the number of alleles and frequency. The PIC values were determined by the expression:

$$PIC=\sum_{i=1}^{n}f_{i}^{2}=1\sum_{j=i+1}^{n=i}2f_{i}^{2}\times f_{j}^{2}$$

In the above expression, fi is the frequency of allele i in the population (Lynch and Walsh, 1998). The PIC provides an estimate of the discriminatory power of the locus, considering the number of alleles that are expressed and the relative frequencies of these alleles.

A binary data matrix 0 and 1 was generated from the coding of the presence (1) and absence (0) of polymorphic bands present in the isolates and then intrapopulational genetic diversity analyses calculated through the POPGENE (Yeh *et al.*, 1997) were performed. Genetic diversity was inferred through the allelic richness and hierarchical analysis of the *Fop* isolates. The values of genetic diversity were estimated based on the number of haplotypes in relation to the total number of isolates and their subpopulations (location). To verify the genetic diversity within and between populations, the following were calculated: Total heterozygosity (H_T_); Mean heterozygosity within the population (Hs), population differentiation coefficient (G_ST_). The estimates of the number and frequency of haplotypes, AMOVA and Wright F_ST_ estimator (1978) were performed using the ARLEQUIN 3.1 software (Excoffier *et al.*, 2005).

### Genome synteny and functional annotation

Molecular markers were aligned to the *Fusarium oxysporum* f. sp. *lycopersici* strain 4287 (Ma *et al.*, 2010) using the native nucleotide basic local alignment search tool (BLASTn) and default algorithm parameters (threshold E-value < 1 × 10^-10^) from JGI (The Fungal Genomics Resource) version 1.0 (https://genome.jgi.doe.gov/Fusox1/Fusox1.home.html).

### Data analysis

The genetic structure of the sample was investigated using the Bayesian clustering algorithm implemented by Structure v.2.3.4 (Pritchard *et al.*, 2000). The No Admixture model was used on the whole dataset with no previous population information and the “no-correlated allele frequencies between populations” option. Ten runs were applied with a burn-in of 200,000 interactions and a run length of 500,000 iterations performed for several clusters varying from K=1 to K=6. To determine the most probable number of clusters, the ad hoc statistic ΔK defined by Evanno *et al.* (2005) was used. The mean of the absolute values of L’(K) was divided by the standard deviation, where L (K) stands for the mean likelihood plotted over ten runs for each K. A hierarchical analysis of variance was carried out to test the significance of the differentiation among the populations and clusters as defined by Structure software.

Dendrogram trees were produced using genotyping data with 14 SSRs markers using the unweighted neighbor-joining method, as implemented in the DARwin software (version 6.0.9).

## Results

From 384 clones that showed good sequencing quality for library 1 (American race), 175 microsatellites were from the American race and from these, 61 clones did not have microsatellites, representing an enrichment of 54.2%. From the total microsatellites found for the American race, dinucleotides were the most abundant (82 sequences), followed by trinucleotides (72 sequences); and among the motifs, the AG dinucleotides were in greater number, followed by the trinucleotide TCA. For the Brazilian race, the most abundant motifs were also the dinucleotides (52 sequences), followed by trinucleotides (48 sequences); and among the motifs, GAG and GAT trinucleotides were found in a greater number, followed by AC dinucleotides (Table 2, Figure S1).

The perfect microsatellites presented the highest number (97 sequences), followed by the compounds (63 sequences) and the imperfect ones (15 sequences) for the American race library. For the Brazilian race library, the perfect microsatellites also presented the highest number (70 sequences), followed by the compounds (35 sequences) and the imperfect ones (11 sequences).

**Figure 1:**
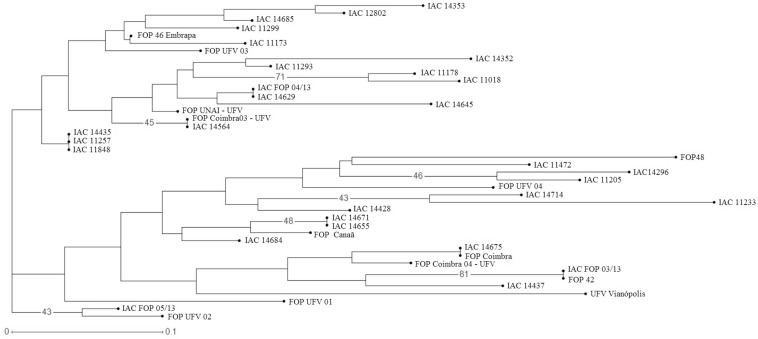
Dendrogram generated using genotyping data with 15 SSRs using the unweighted neighbor-joining method, as implemented in the DARwin software. Bootstrap node support was represented in percentages and showed clustering stability. Numbers (%) on the branches correspond to bootstrap values above 45%.

A total of 40 SSRs were developed, and out of these, 15 SSRs were polymorphic (Table 2). The number of alleles ranged from 2 to 10, with an average of four alleles per *locus*. The highest PIC (Polymorphic Information Content) value was 0.77 for Fop10-B01 (Table 2). The average PIC found was 0.38. In the genetic diversity analysis (Figure 1), the isolates were separated into two major clusters; each of these groups was divided into two smaller subgroups.

**Figure 2:**
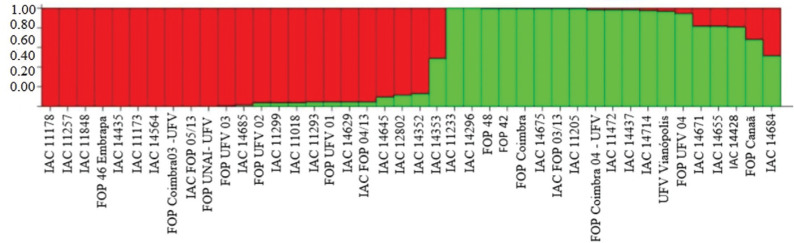
The genetic structure of the *Fop* isolates was investigated using the Bayesian clustering algorithm implemented by STRUCTURE v.2.3.4

Population analysis performed by Structure software also reinforced these results (Figure 2). The best results were obtained at K = 2 (Figure S2) by Evanno *et al.* (2005). There was a great correspondence between the isolates grouped by clustering analysis and by population structure analysis except for *Fop* UFV 01, IAC *Fop* 05/13 and *Fop* UFV 02, which grouped externally in the dendrogram and fitted in the first group of Structure.

The percentage of polymorphic *loci* in isolates within each state ranged from 4.17 to 84.32%, showing low genetic variability of the isolates collected in the states of Goiás (15.62%), Pernambuco (13.54%), Minas Gerais (34%), Santa Catarina (14%) and Paraná (4.17%). The isolates derived from the state of São Paulo presented the highest genetic variability with 84% polymorphic *loci* and the isolates from the state of Paraná presented the lowest genetic variability with 4.17% polymorphic *loci* (Table S1).

The estimated total heterozygosity (H_T_) was intermediate (Table 3). The mean value obtained from F_ST_ (0.12) and G_ST_ (0.33), indicated the genetic structure in the total sample (Table 3). The distribution of genetic variability between and within Fop isolates was characterized by the genetic divergence of Nei (1978). The results of the AMOVA showed that 12% of the genetic variability was among the six States and 87% was within each state (Table 4).

**Table 3:** Mean and standard deviation of the genetic parameters of the *Fusarium oxysporum* f. sp. *phaseoli* isolates.

	H_T_	H_S_	G_ST_	F_ST_
Mean	0.13	0.09	0.33	0.12
Standard deviation	0.02	0.009

H_T_: total heterozygosity; H_S_: mean gene diversity; G_ST_: gene divergence among isolates; F_ST_: inbreeding among isolates.

**Table 4:** Molecular variance analysis (AMOVA) of *Fusarium oxysporum* f. sp. *phaseoli* microsatellites.

Populations	DF*	MSSs**	Variance Components	% of the variance
Among isolates	04	19.58	0.30	12.09
Within isolates	38	84.05	2.21	87.91
Total	42	103.64	2.52

* DF – Degrees of Freedom;

** MSSs – mean sum of squares

Blasting of the expected sequence amplified by the SSRs against *Fusarium oxysporum* f. sp. *Lycopersici* genome identified sequences highly similar in chromosomes 1, 2, 4, 5, 7 and 8 (Table 5).

**Table 5:** Annotation of the SSRs on *Fusarium oxysporum* f. sp. *phaseoli* isolates.

Marker name	Organism	Chr^a^	*E-value*	Gene symbol	Functional annotation^b^
FOP 01-B01	*Fusarium oxysporum f. sp. lycopersici*	5	0.0	FOXG_15195	Hypothetical protein
FOP 07-B01	*Fusarium oxysporum f. sp. lycopersici*	1	0.0	FOXG_00881	Cysteine synthase A
FOP 10-B01	*Fusarium oxysporum f. sp. lycopersici*	2	3e-164	FOXG_08277	26S proteasome regulatory subunit rpn-1
FOP 13-B01	*Fusarium oxysporum f. sp. lycopersici*	7	1e-73	FOXG_04761	Hypothetical protein
FOP 15-B01	*Fusarium oxysporum f. sp. lycopersici*	4	0.0	FOXG_07953	Hypothetical protein
FOP 16-B01	*Fusarium oxysporum f. sp. lycopersici*	2	8e-155	FOXG_08472	Hypothetical protein
FOP 20-B01	*Fusarium oxysporum f. sp. lycopersici*	2	0.0	FOXG_11588	Hypothetical protein
FOP 28-B01	*Fusarium oxysporum f. sp. lycopersici*	2	1e-107	FOXG_08344	Nudix_Hydrolase
FOP 35-B01	*Fusarium oxysporum f. sp. lycopersici*	8	0.0	FOXG_18524	Hypothetical protein
FOP 37-B01	*Fusarium oxysporum f. sp. lycopersici*	7	4e-99	FOXG_05342	Hypothetical protein
FOP 08-B2	*Fusarium oxysporum f. sp. lycopersici*	1	0.0	FOXG_01377	Hypothetical protein
FOP 09-B02	*Fusarium oxysporum f. sp. lycopersici*	Unknown	0.0	FOXG_17405	Hypothetical protein
FOP 11-B02	*Fusarium oxysporum f. sp. lycopersici*	2	0.0	FOXG_08568	Ribokinase
FOP 18-B02	*Fusarium oxysporum f. sp. lycopersici*	1	0.0	FOXG_11114	Hypothetical protein
FOP 23-B02	*Fusarium oxysporum f. sp. lycopersici*	5	0.0	FOXG_01709	Hypothetical protein

## Discussion

*Fusarium oxysporum* f. sp. *phaseoli* (Fop) occurs in almost all common bean Brazilian fields (Toledo-Souza *et al.*, 2012), reducing the yield significantly. In the case of Fop, many questions are still open, especially in what concerns the amount of diversity present within this *forma specialis*. Accurate and rapid identification of Fop is a needed for appropriate disease management. DNA-based techniques have increasingly become the tool of choice for understanding the genetic diversity and phylogeny relationships of *Fusarium* spp. (Huang *et al.*, 2013; Cruz *et al.*, 2018; Petkar *et al.*, 2019). Microsatellites have been used because of the high resolution they provide (Dutech *et al.*, 2007; Cruz *et al.*, 2018); however, obtaining an acceptable level of polymorphism is generally more difficult in fungi than in other organisms (Dutech *et al.*, 2007). According to Gao *et al.* (2008), most SSRs found in fungi are dinucleotides and trinucleotides, located in non-coding regions of the genome. This is in accordance to what we have found in both Brazilian and American race libraries. The size of the fungi genome (~59.9 Mb for *Fusarium oxysporum*) is smaller than those of plants and the longer the genome sequence, the longer the repeated units (Hancock, 2002). Indeed, the relative abundance of SSRs in fungi is low compared with the human genome, and long SSRs in fungi are rare (Karaoglu *et al.*, 2005)

The genetic diversity among the isolates of *Fop* in this study was low which is in accordance to literature (Bogale *et al.*, 2006). The *Fusarium oxysporum* species presents asexual reproduction and does not present variation due to meiotic recombination (Ming *et al.*, 1966). Cruz *et al.* (2018) showed that there was no relationship between the location and the genetic groups found by SSRs clustering. In our study, both the dendrogram and the Bayesian analysis showed that differences between the isolates were not related to their geographical origin. In fact, the introduction of the pathogen into different areas occurs mainly by infected seeds (Fonseca *et al.*, 2002). Common bean seeds of the previous crop are used for planting in the next crop, usually without control of the phytosanitary quality. The exchange and trade of these seeds is a common practice among farmers. Therefore, the absence of strong genetic structure at different spatial scales might be likely related to the spread of the pathogen through human activities. In addition to the nonrandom distribution of SSR clusters in the genome (Vieira *et al.*, 2016), *Fop* transfer of specific genomic regions (Ma *et al.*, 2010) might contribute to this low isolation by location. In relation to the intermediate estimated total heterozygosity (H^T^, Table 3), the species seems to have a reasonable reserve of genetic variability and the AMOVA showed higher genetic variability within each state (Table 4). In fact, Cruz *et al.* (2018) used SSRs to characterize the genetic diversity of *F. oxysporum* collected from common bean in different Brazilian states and reported that it was not possible to group them by collected location, or by their pathogenicity.

One of the problems in the characterization of isolates in the *F. oxysporum* species complex is the assumption that there is a link between pathogenicity and a specific host or a group of host species and sub-species taxa. In most cases this assumption is either incorrect or an oversimplification of the actual situation (Bogale, 2006). Non-pathogenic *F. oxysporum* isolates are genetically diverse and make up a significant component of the species complex (Bao *et al.*, 2002). Moreover, many phylogenetic studies have shown that some pathogenic isolates are more closely related to non-pathogenic strains than to other pathogenic strains in the same *formae* speciales or races (Mohammadi *et al.*, 2004; Pasquali *et al.*, 2004). This suggests that pathogenicity may be governed by a few genes, and that most likely, pathogenic genotypes in *F. oxysporum* arise by transfer of “pathogenicity chromosomes” (Ma *et al.*, 2010). Consequently, the use of pathogenicity as a sole characteristic for grouping *F. oxysporum* isolates is flawed (Skovgaard *et al.*, 2002).

The average marker's polymorphic information content (PIC) was higher than the one reported by Cruz *et al.* (2018) and much alike to the one reported by Bogale *et al.* (2005), who found an allelic diversity for nine microsatellite *loci* of 0.003-0.895 and a total of 71 alleles among 64 *F. oxysporum* isolates.

According to the annotation performed, it was found homology to a nudix hydrolase (FOP28-B01, Table 5), which constitutes a large family of proteins that hydrolyze nucleoside diphosphate derivatives. Nudix effectors have been reported in plant pathogenic oomycetes, fungi, and bacteria suggesting that these effectors might be important virulence components in the “toolbox” of plant pathogens (Ge and Xia, 2008; Dong and Wang, 2016).

The Fop10-B01 sequence had high homology with *Fusarium oxysporum* f. sp. *lycopersici* 4287 26S proteasome regulatory subunit RPN-1 mRNA (99% identity, E-value 3e-164, Table 5). RPN1a is required for basal defense and R protein-mediated defense (Yao *et al.*, 2012). Some subunits of the 26S proteasome were found to be involved in innate immunity in Arabidopsis and probably in *Fusarium oxysporum* too.

In Brazil, *Fusarium oxysporum* f. sp. *phaseoli* (*Fop*) occurs in almost all common bean-producing areas (Batista *et al.*, 2016), significantly reducing the yield. The difficulty in obtaining resistant genotypes to *Fop* is due to the diversity of physiological races that the pathogen presents, making necessary the study of the genetic and physiological variability of the pathogen. Several control methods are available, but none are efficient. There is no completely resistant common bean cultivar available on the market and no dominant resistance gene has been properly identified yet. The development of tools allowing early detection of the pathogen and effective disease control relies on the knowledge of the pathogen diversity. This study is expected to serve as an important groundwork for further genetic variation research on *F. oxysporum* f. sp. *phaseoli*.

## Supplementary Material

The following online material is available for this article:

Figure S1 - Electropherogram with the peak of the allele of the microsatellite.

Figure S2 - Number ideal cluster's according to the methodology of EVANO *et al.* (2005).

Table S1 - Mean, standard deviation of the genetic parameters obtained for the 14 microsatellites.
