# Supplementary material for: Fusarium oxysporum f. sp. phaseoli genetic variability assessed by new developed microsatellites
Source: Genet Mol Biol. 2020 May 29;43(2):e20190267. doi: 10.1590/1678-4685-GMB-2019-0267 (PMC7263423; doi:10.1590/1678-4685-GMB-2019-0267)
Supplement: Supplementary file 1 [file 1415-4757-GMB-43-2-e20190267-s1.pdf]

Supplementary Material to "*Fusarium oxysporum* f. sp. *phaseoli* genetic variability assessed by new developed microsatellites"

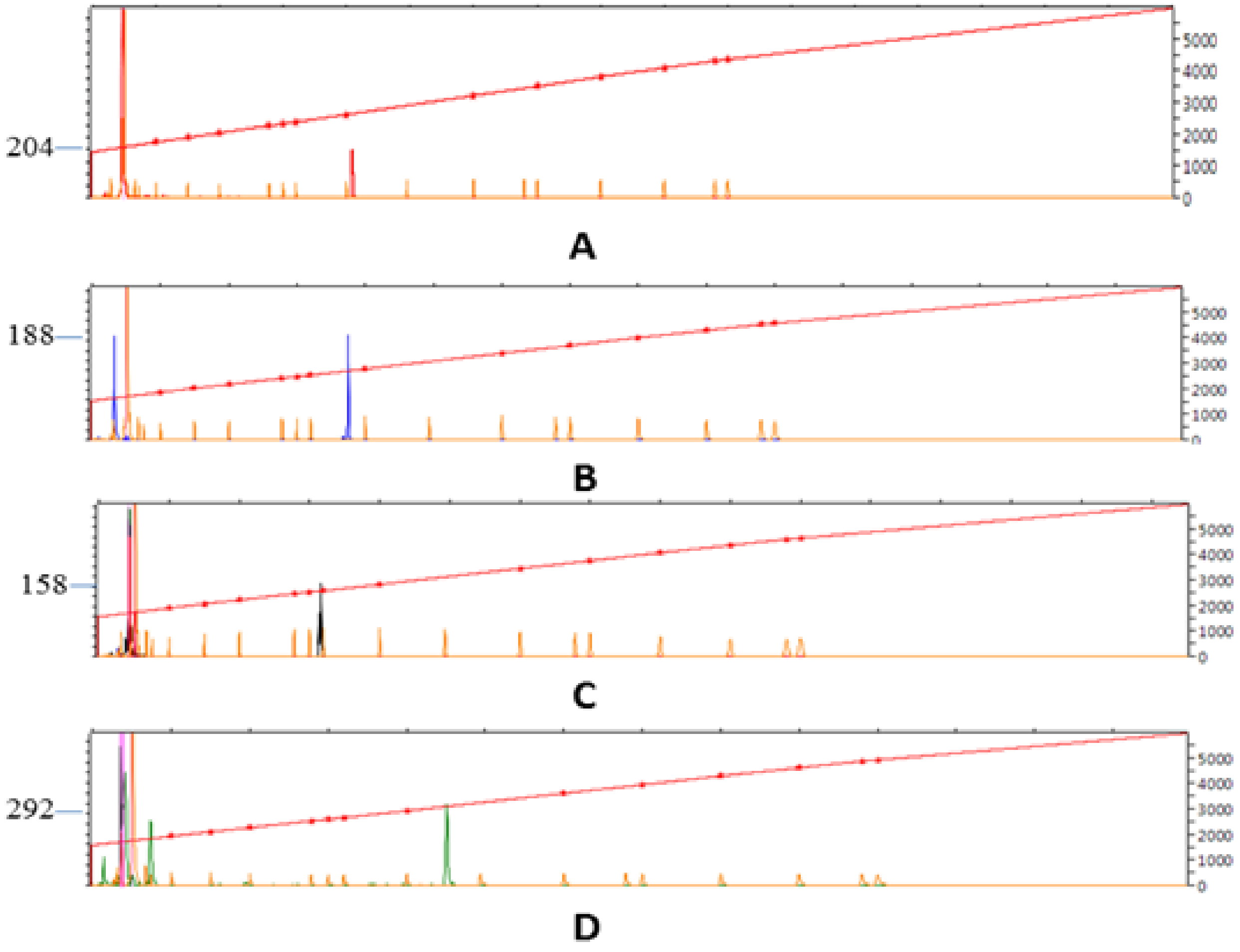

Figure S1. Electropherogram with the peak of the allele of the microsatellite markers: A. FOP 28-B01 with flowering PET (red staining), B. FOP 37-B01 with blue fluorescence (6-FAM), C. FOP 04-B01 with black fluorescence (NED) and D. FOP 11-B02 with green fluorescence (VIC). The orange colored peaks are from the "LIZ 500" (molecular weight standard). Peak Scanner TM v.1 program (Applied Biosystems).
