# Supplementary material for: Fusarium oxysporum f. sp. phaseoli genetic variability assessed by new developed microsatellites
Source: Genet Mol Biol. 2020 May 29;43(2):e20190267. doi: 10.1590/1678-4685-GMB-2019-0267 (PMC7263423; doi:10.1590/1678-4685-GMB-2019-0267)
Supplement: Supplementary file 2 [file 1415-4757-GMB-43-2-e20190267-s2.pdf]

Supplementary Material to "*Fusarium oxysporum* f. sp. *phaseoli* genetic variability assessed by new developed microsatellites"

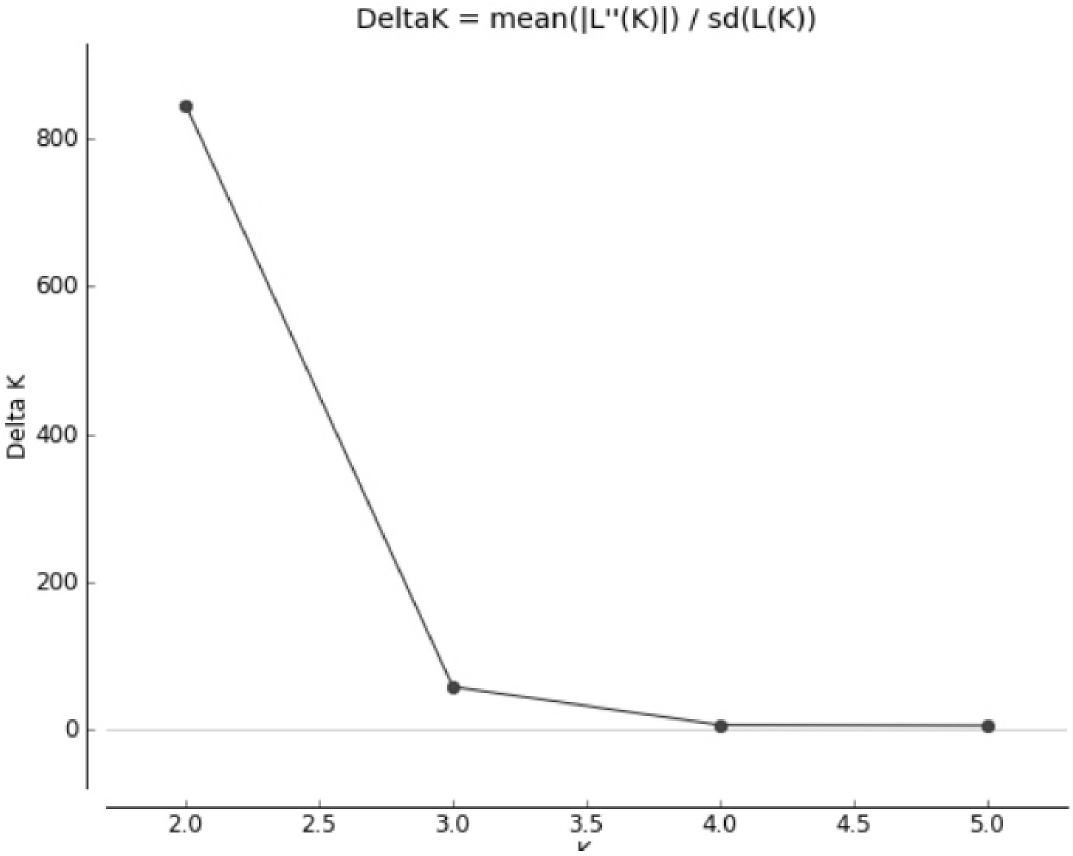

Figure S2. Number ideal cluster's according to the methodology of Evanno *et al*. (2005).
