# Supplementary material for: Fusarium oxysporum f. sp. phaseoli genetic variability assessed by new developed microsatellites
Source: Genet Mol Biol. 2020 May 29;43(2):e20190267. doi: 10.1590/1678-4685-GMB-2019-0267 (PMC7263423; doi:10.1590/1678-4685-GMB-2019-0267)
Supplement: Supplementary file 3 [file 1415-4757-GMB-43-2-e20190267-s3.pdf]

Supplementary Material to "*Fusarium oxysporum* f. sp. *phaseoli* genetic variability assessed by new developed microsatellites"

Table SS. Mean, standard deviation of the genetic parameters obtained for the 14 microsatellites that amplified the isolates of *F. oxysporum* f. sp. *phaseoli* collected from the states of Goiás, São Paulo, Pernambuco, Minas Gerais, Santa Catarina and Paraná of the Federative Republic of Brazil.

Â: number of alleles; ne: effective population size; H_E: Nei genic diversity; i: Shannon´s genetic diversity; P%: percent of polymorphic loci; s: standard deviation.

**Parameters**	**Isolates**													
	**GO**		**SP**		**PE**		**MG**		**SC**		**PR**		**Total**	
**Â**	1.,16	(0.37)	1.84	(0.36)	1.13	(0.34)	1.34	(0.48)	1.15	(0.36)	1.04	(0.20)	2.00	(0.00)
**Ne**	1.14	(0.32)	1.30	(0.32)	1.10	(0.27)	1.15	(0.26)	1.15	(0.36)	1,00	(0.20)	1.24	(0.27)
**H_E**	0.07	(0.17)	0.19	(0.16)	0.06	(0.15)	0.10	(0.15)	0.07	(0.18)	0.02	(0.10)	0.16	(0.14)
**I**	0.10	(0.24)	0.31	(0.22)	0.08	(0.22)	0.16	(0.23)	0.01	(0.25)	0.03	(0.14)	0.28	(0.19)
**P%**	15.62	-	84.32	-	13.54	-	34	-	14	-	4,17	-	27.60	-
